# Stereotactic Radiotherapy: An Alternative Option for Refractory Ventricular Tachycardia to Drug and Ablation Therapy

**DOI:** 10.3390/jcm11123549

**Published:** 2022-06-20

**Authors:** Wenfeng Shangguan, Gang Xu, Xin Wang, Nan Zhang, Xingpeng Liu, Guangping Li, Gary Tse, Tong Liu

**Affiliations:** 1 Tianjin Key Laboratory of Ionic-Molecular Function of Cardiovascular Disease, Department of Cardiology, Tianjin Institute of Cardiology, Second Hospital of Tianjin Medical University, Tianjin 300211, China; sgwf@163.com (W.S.); xugang_163com@163.com (G.X.); 13502076469@163.com (X.W.); zhangnancardiac@126.com (N.Z.); tjcardiol@126.com (G.L.); 2 Department of Heart Center, Beijing Chaoyang Hospital, Capital Medical University, 8th Gongtinanlu Rd., Chaoyang District, Beijing 100020, China; xpliu71@hotmail.com; 3 Faculty of Health and Medical Sciences, University of Surrey, Guildford GU2 7XH, UK; 4 Kent and Medway Medical School, Canterbury CT2 7FS, UK

**Keywords:** stereotactic body radiotherapy, refractory ventricular tachycardia, treatment

## Abstract

Refractory ventricular tachycardia (VT) often occurs in the context of organic heart disease. It is associated with significantly high mortality and morbidity rates. Antiarrhythmic drugs and catheter ablation represent the two main treatment options for refractory VT, but their use can be associated with inadequate therapeutic responses and procedure-related complications. Stereotactic body radiotherapy (SBRT) is extensively applied in the precision treatment of solid tumors, with excellent therapeutic responses. Recently, this highly precise technology has been applied for radioablation of VT, and its early results demonstrate a favorable safety profile. This review presents the potential value of SBRT in refractory VT.

## 1. Background

Refractory ventricular tachycardia (VT) is characterized by recurrent sustained VT despite medical or interventional treatment. Antiarrhythmic drugs have been used to treat VT for many years [1], but they are ineffective in some cases, and their use can be associated with side effects [2,3]. Catheter ablation utilizes radiofrequency or cryo-energy to destroy the scarred cardiac tissue and its surrounding tissue, effectively disrupting the re-entrant VT substrate [4,5]. Catheter ablation can be more effective than antiarrhythmic drugs [4] but may fail due to its inability to reach the arrhythmogenic target tissue or deliver adequate energy to the deep target myocardium-tissue from which the VT originates. Catheter ablation application is also limited by the occurrence of transmural injury. The recurrence rate after VT catheter ablation is as high as 50% [6]. Catheter ablation may also be associated with procedural risks and mortality [7]. An implantable cardioverter-defibrillator (ICD) can effectively terminate VT or ventricular fibrillation, reducing the risk of sudden death [8]. However, recurrent ICD shocks are painful, contribute to impaired quality of life [9,10], and are associated with higher mortality and worsening of heart failure [11,12].

## 2. Stereotactic Body Radiotherapy

Stereotactic body radiotherapy (SBRT) is mostly used to treat solid tumors [13]. Unlike traditional radioablation, SBRT employs radiation delivered to a 3D target volume created by various imaging technologies, accurately ablating the target volume while avoiding damage to the surrounding tissue, with up to submillimeter accuracy. Early findings reported that this noninvasive technology could effectively treat refractory VT. Unlike radiofrequency or cryo-energy ablation, SBRT is noninvasive and painless. SBRT targeting of the VT substrate of origin is an alternative for patients with refractory VT after several failed catheter ablations.

## 3. Mechanisms of SBRT

The exact mechanisms through which radioablation injures the target myocardium during refractory VT treatment have not been fully characterized. Some proposed radiotherapy injury mechanisms include double-strand DNA breaks that lead to cell apoptosis, vascular injury that induces tissue hypoxia, and ischemia-related cell death. Kiani [14] described the cellular effects of radiotherapy on the human myocardium. Histopathologic features were indicative of cell injury, death, and fibrosis; electron microscopy demonstrated features consistent with disruption of the cardiomyocyte architecture and cellular machinery. Indirect radiotherapy mechanisms related to tissue vasculature damage, cell hypoxia, and necrosis induce myocardial fibrosis and conduction block that may prevent further arrhythmic events [15,16].

Previous studies speculated that radioablation induced transmural fibrosis and a homogeneous myocardium, which might account for the reduced VT after SBRT. Heart radioablation with a dose of 25 Gy or higher in animal studies produced target myocardial-tissue degeneration and fibrosis and full transmural heart-injury months after the treatment [17]. Sharma et al. [18] found that radiotherapy with a single dose of at least 25 Gy could produce cavotricuspid isthmus and atrioventricular (AV) nodal blocks, and alter the electrophysiological properties at the pulmonary vein–left atrial junction. Blanck et al. [19] reported that a radioablation dose above 32.5 Gy to a healthy pig heart can induce transmural heart injury six months after treatment. Refaat et al. [20] reported that AV conduction block was induced by radioablation at a dose of 35–40 Gy, showing myocardial fibrosis in the AV node radioablation target area using immunostaining. Patients treated with a single radioablation dose of 25 Gy showed a reduced VT burden within days [21] or weeks after treatment [19,22]. Therefore, radiotherapy-induced myocardial fibrosis cannot fully explain the early effect of radioablation. An animal study found no apparent myocardial necrosis and apoptosis one month after a high-dose cardiac radioablation, further supporting the notion that fibrosis cannot explain the early antiarrhythmic effects of such a treatment [23].

Zhang et al. [24] presented evidence in an animal model and in patients that cardiac radioablation induced electrical conduction reprogramming rather than causing transmural fibrosis during the early period after treatment. Six weeks after a 25-Gy radioablation was delivered to murine hearts, no significant myocardial fibrosis or increased collagen content was detected. However, cardiac-electrophysiology remolding was found, with a significantly shortened QRS interval and increased ventricular conduction velocity. These results showed that 25-Gy radioablation induced cardiac-electrophysiology remolding without transmural fibrosis. This explains why the SBRT effect was observed within days to weeks. Western blots showed a significant increase in the expression of NaV1.5 (the cardiac voltage-gated sodium channel subunit that plays a key role in phase-0 depolarization of the action potential) and connexin 43 (a major subunit of the ventricular gap junction that helps pulse diffusion) after radioablation in murine hearts. The high expression of NaV1.5 and connexin 43 was present even 42 weeks after radioablation, achieving persistent cardiac electrical reprogramming. The Notch signaling pathway plays an important role in radioablation-induced cardiac electrophysiology reprogramming. Kim et al. [25] explored the immediate effect of radioablation on myocardial cells, showing that high-dose radioablation regulated cardiomyocyte electrophysiological activities immediately, possibly accounting for the immediate antiarrhythmic effect of SBRT on refractory VT. Additionally, animal experiments showed that radiation increased connexin 43 expression, improved the myocardial conduction velocity, reduced repolarization spatial heterogeneity, and decreased the risk of ventricular arrhythmias after myocardial infarction [26]. We can see from the above studies that radioablation-induced myocardial electrophysiology remolding, rather than fibrosis, accounted for the early effect of VT radiotherapy. The exact effects of radiation on the biology and electrophysiologic properties of the myocardium need further research.

## 4. SBRT Planning and Implementation

Unlike radiotherapy for tumors, the radioablation target in VT is defined by electrical properties and cannot be accurately displayed by imaging alone. Therefore, SBRT requires a multi-disciplinary team that includes oncologists, medical physicians, and cardiac electrophysiologists. Cardiac electrophysiologists can identify potential candidates and localize the re-entrant substrate using electroanatomic or other noninvasive mapping techniques. These details could assist medical physicians and oncologists to create an accurate 3D target volume, transferring these data into the SBRT system, and formulating and implementing VT radiotherapy plans.

The first VT radioablation step is to precisely identify the substrate causing the VT, often related to the underlying structural heart disease. In scar-related VT, the arrhythmia often originates from ventricular scar border zones [27]. In cases with non-ischemic causes such as dilated cardiomyopathy, the arrhythmogenic substrate often involves fibrotic areas in the myocardium and has a dispersed distribution. However, only part of the scar volume contributes to VT generation. Some surviving cardiac fibers within and around the scar tissue create slow conduction pathways and form the basis for the re-entrant VT-generating substrate. Therefore, an extensive substrate ablation that homogenizes the scar would improve the ablation success rate [28]. The myocardial scar tissue can be identified using delayed-enhanced magnetic resonance imaging (MRI), positron emission tomography–computed tomography (PET-CT), nuclear perfusion imaging, or echocardiography. Most patients with refractory VT have undergone 3D electroanatomic mapping and an unsuccessful catheter ablation procedure before cardiac SBRT. Noninvasive arrhythmia substrate mapping before SBRT would help define and localize the ablation targets [29].

Precise targeting of the arrhythmogenic substrate is crucial in VT radioablation, as it could help reduce off-target ablation of surrounding healthy myocardium, organs, and implantable electronic devices. Accurately transforming the substrate information to target volume planning is a difficult task. Multimodal integration of the electroanatomic mapping with computed tomography (CT) planning could help accurately localize the target on the CT images [30]. High-quality endocardial-surface and -chamber mapping could help to precisely merge the information with cardiac CT [31]. The RAVENTA trial found remarkable differences between the electroanatomic mapping outcomes and target volume planning during VT radioablation [32]. Therefore, highly effective and uniform standards or guidelines are required to transfer the electroanatomic mapping information to the CT planning.

The heart is a moving target due to its intrinsic spontaneous mechanical activity and motions caused by respiration. Initially, SBRT was thought to be unsuitable for cardiac arrhythmia treatment due to difficulties in localizing a moving target. However, with the great progress in imaging technologies such as the gating and tracking systems, the scope of SBRT has been expanded to include VT treatment [22]. Imaging strategies are used to differentiate the target tissue volume from the surrounding healthy tissue. Motion compensation strategies include dampening or inhibition to restrain respiratory motion, gating to release radiation at a specific time of the respiratory cycle, and tracking to make the radiation beam follow the moving target [33]. Ho et al. [34] reported that computational ECG mapping and a protocol-guided respiratory gating system could improve target volume planning precision, helping with SBRT planning and implementation. Cha et al. [35] proposed a new method, using deep-inspiration breath-hold to separate the target from the stomach, reducing the dose delivered to the gastrointestinal tract during cardiac SBRT. Real-time cardiorespiratory-motion-mitigated MRI could help compensate for cardiac and respiratory motions simultaneously [36]. Although many techniques have been used to minimize injury to the surrounding tissues, it is impossible to fully avoid the off-target delivery of radiation beams due to the respiratory and cardiac motions.

SBRT duration ranges between 10 and 90 min, depending on the radiation dose, the machines used, and the target tissue volume, location, and size (Table 1). Another key issue in SBRT for VT ablation is the radiation dose. Blanck et al. [19] showed, in a pig model, that a dose of 17.5 Gy did not induce any significant fibrosis in pulmonary veins, a dose of 25 Gy induced mild fibrosis, and a dose above 32.5 Gy might lead to transmural fibrosis. Animal study outcomes supported 25 Gy as the radioablation threshold dosage to create target myocardial-tissue fibrosis, but higher radiation dosages (35–40 Gy) increased the risks of radiation-related complications [18,19,37]. Based on preclinical results and experience in oncology SBRT, most clinical studies used 25 Gy as the basic treatment dose. The currently prescribed 25-Gy dose might be insufficient to form a homogenous transmural scar. Zhang et al. [24] proposed that the radioablation mechanism was mediated by electrophysiologic reprogramming rather than myocardial fibrosis. Using a dose de-escalation method, they found that a dose of 5–10 Gy did not induce significant electrophysiologic reprogramming, while a dose higher than 15 Gy increased the ventricular conduction velocity and shortened the QRS interval. The largest electrophysiologic effects were detected at a dose of 25 Gy. Elucidation of the optimal dose regimen needs further study.

## 5. SBRT for VT Ablation: Clinical Experience

To date, a limited number of prospective and retrospective studies or case series focusing on the clinical evidence of SBRT for VT have been published. Loo et al. [38] delivered the first in-human cardiac arrhythmia treatment in 2012 using stereotactic radioablation with a radiation dose of 25 Gy. After a 2-month blanking period, the number of VT episodes remained low for seven months, with no acute or late complications. However, refractory VT occurred nine months after the procedure in the context of an exacerbation of chronic obstructive pulmonary disease, culminating in the patient’s death.

In a case series reported by Cuculich et al. [22], five patients at high risk of refractory VT underwent SBRT with a single delivery dose of 25 Gy and a 14-min mean radioablation duration. The pre-procedural VT burden of these patients was 6577 episodes over a 3-month time period. During the 6-week post-ablation blanking period, the VT episodes decreased to 680 and declined to 4 over the next 46 patient-months. All five patients presented with a significant reduction in VT burden from the baseline. The mean left-ventricular-ejection fraction was preserved throughout the follow-up.

In 2019, Robinson et al. [40] published a phase I-II prospective trial of VT radioablation. Nineteen patients with treatment-refractory VT or premature-ventricular-contraction (PVC) cardiomyopathy were enrolled and received noninvasive electrophysiology-guided cardiac radioablation with a single delivery of 25 Gy. The number of VT episodes decreased from 119 to 3 in 18 patients after the ablation, 17 of which (94%) presented with reduced VT or PVC burden. The overall 6- and 12-month survival rates were 89% and 72%, respectively; the dual antiarrhythmic drug use declined from 59 to 12%; and the quality of life at six months improved in five of the nine Short Form-36 domains. Evidently, noninvasive electrophysiology-guided cardiac radioablation could bring favorable clinical benefits to patients with VT or PVC-related cardiomyopathy.

Neuwirth et al. [39] reported a case series of ten patients with structural heart disease and refractory VT. They delivered 25 Gy to the planning target volumes using CyberKnife. Compared to the baseline, the VT burden significantly decreased by 87.5% during the 28-month follow-up. Three patients died of non-arrhythmic causes. After a blanking period of 90 days, eight of the patients experienced a recurrence of VT. The mean times from treatment to the first antitachycardia pacing and ICD shock were 6.5 and 21 months, respectively. The follow-up results showed that SBRT failed in two patients, and two patients showed a delayed response of three and six months, respectively. One patient with previously known mitral regurgitation showed progression of the regurgitation and valvular morphology changes 17 months after the radioablation procedure. This study showed that SBRT was a safe and effective long-term treatment for refractory VT and could improve the survival rate of these patients.

Gianni et al. [42] described five patients with structural heart disease and refractory VT. A single-fraction radiation dose of 25 Gy was delivered to the target volume using CyberKnife at a mean treatment duration of 82 min. Four of the five patients presented with significant reductions in the number of VT episodes and in the use of antiarrhythmic drugs during the first six months after the ablation. All five patients experienced VT recurrence during the 12-month follow-up, of which two died from heart failure worsening. Those researchers found no evidence that the radioablation injured the cardiac tissue around the target volume. Despite the initial promising results, SBRT did not show long-term effectiveness in patients with refractory VT. No radiation-related complications were noted during follow-up, suggesting an acceptable safety profile for SBRT. Further studies are needed to elucidate the mechanisms associated with the lack of long-term efficacy.

Lloyd et al. [41] reported ten patients with advanced heart failure (four with ischemic and six with non-ischemic cardiomyopathy) and refractory VT who underwent SBRT. The mean patient age was 61 years. The results showed that the number of VT episodes decreased by 69%, antitachycardia pacing sequences decreased by 48%, and ICD shocks decreased by 68% after the SBRT. One patient in this study did not respond to the SBRT. After excluding this patient, a significant decrease in the VT burden was observed after treatment.

Chin et al. [43] retrospectively analyzed eight consecutive patients with refractory VT who underwent SBRT, with an average age of 75 years and a mean ejection fraction of 21%. All patients were male, half with ischemic and half with non-ischemic cardiomyopathy. A mean dose of 22.2 Gy was delivered to the planning target volume during a single radioablation treatment session. During the 7.8-month follow-up, the number of ICD shocks decreased from 69.5 to 13.3. No acute complications or definite peripheral organ toxicities occurred during follow-up. Three patients died during follow-up, of causes unrelated to the SBRT. This patient group experienced VT burden reduction after the SBRT, but, unlike previous reports, they showed no immediate effects.

Carbucicchio et al. [44] presented the preliminary results of their spontaneous, prospective, single-arm, phase Ib/II single-center STRA-MI-VT Study. The study investigated the safety and efficacy of SBRT in eight patients with intractable VT. Of the seven patients who underwent SBRT (mean age, 70 ± 7 years; ejection fraction, 27 ± 11%), three had ischemic and four had non-ischemic cardiomyopathy. No treatment-related serious adverse events occurred at a median follow-up of eight months. Three patients died from non-SBRT-related causes, while four completed the 6-month follow-up. The number of VT episodes decreased from 29 to 11 and 2, three and six months post-treatment, respectively. The respective number of ICD shocks decreased from 11 to 0 and 2. All patients showed a significant reduction in the number of VT episodes and no electrical storm recurrence six months after treatment. The STRA-MI-VT Study showed excellent short-term VT radioablation treatment outcomes but showed no long-term effects.

The study by Qian et al. [45] aimed to determine the feasibility of using radioablation on the arrhythmogenic substrate of myocardial scars in patients with ischemic cardiomyopathy. Six male patients with ischemic cardiomyopathy and refractory VT underwent SBRT targeting of the extensive scar substrate. The median planning target volume was 319 mL, and the radioablation dose was 25 Gy. ICD-treated or sustained VT episodes did not decrease significantly after SBRT, but ICD shocks decreased significantly from 12 to 0. Three patients died of heart failure during the 7.7-month follow-up period, and three experienced complications, including heart failure exacerbation, pneumonia, and asymptomatic pericardial effusion. Repeat catheter ablation was performed in four patients at 32, 167, 288, and 396 days post-SBRT. This study suggested that SBRT-induced substrate modifications did not significantly reduce the VT burden in patients with ischemic cardiomyopathy, similar to a recent report demonstrating that radioablation did not have long-term effectiveness in patients with serious ischemic cardiomyopathy [38]. Further study is warranted to evaluate the radiobiology of myocardial scars, optimal radiation dose, target location, and the effectiveness and safety of refractory VT radioablation in patients with ischemic cardiomyopathy.

Ho et al. [34] enrolled six consecutive patients with refractory VT managed by SBRT. The VT origin sites were identified noninvasively by a 3D computational electrocardiogram (ECG) algorithm and compared to available electroanatomic maps. A 25-Gy radioablation was delivered to the target at the end of expiration. Respiratory gating facilitated small planning target volumes and prevented gastrointestinal complications. ICD shocks decreased from 23 to 0.67 per patient six months post-SRBT. This study showed that a workflow that combined computational ECG mapping and protocol-guided respiratory gating was effective and safe, and could improve SRBT planning.

However, long-term follow-ups indicate a high VT recurrence rate in patients with refractory VT after SRBT, the reasons for which remain unknown. Gianni et al. [42] described three patients with recurrent VT after radioablation. Voltage mapping during repeated radiofrequency catheter ablation showed remaining low voltage, fractionated electrograms within the radioablation target regions. Some surviving cardiomyocytes in the scar suggested incomplete homogenization. Therefore, the 25-Gy radiation dose used in previous studies might be insufficient to destroy the entire target tissue. The uniform delivery of a 25-Gy radiation dose, regardless of the planning target volume, size, and characteristics, needs further exploration and study. Another possibility is that the identified substrate might not be very accurate, as location discrepancies were introduced when the data were transferred to the CT imaging system [32]. Furthermore, aggravation of the myocardial ischemia or exacerbation of the heart failure might produce a new VT substrate, especially in patients with severe ischemic cardiomyopathy. The findings of the many ongoing clinical trials will be able to address some of these issues.

## 6. Safety and Complications

Several studies have reported that the VT radioablation treatment was safe during short-to-medium follow-ups, with only a few complications reported. These included pericarditis (*n* = 1), the progression of valvular disease (*n* = 1), self-resolving pneumonitis (*n* = 2), and delayed pericardial effusion (*n* = 5) [39,40]. There have been no reports of VT radioablation treatment as the direct cause of death (Table 1). Longer-term results from the Phase I/II ENCORE-VT Study showed one serious Grade 4 complication (gastro-pericardial fistula) at 2.4 years requiring surgical repair [52]. Haskova et al. [53] reported a refractory VT patient who died because of a bleeding esophago-pericardial fistula six months after a 25-Gy radioablation. To date, not many patients have undergone SBRT, so two fistula cases suggest that this fatal complication is not uncommon. Nevertheless, we may gain some insights from cancer radiotherapy. Several studies showed a significant relationship between the delivered cardiac radiation doses and the long-term (several years or even decades) cumulative incidence of cardiac events in the coronary arteries, conduction system, valvular structures, myocardium, and pericardium [54]. We need to be alert to late complications such as esophago-pericardial fistula. Long-term follow-up is needed to fully identify potential late complications.

## 7. Limitations

The following limitations of SBRT should be taken into consideration. First, it requires accurate identification and localization of the substrate. Second, an optimal radiation dosing regimen is still lacking and should be further elucidated. Third, the long-term safety and efficacy of SBRT remain unknown. Therefore, SBRT should be reserved for patients with refractory VT following failed medical therapy and ablation.

## 8. Conclusions

SBRT represents a noninvasive option for cases of refractory VT with failed antiarrhythmic therapy and ablation. Further clinical trials and registry studies are needed to better inform and optimize its treatment parameters and characterize its long-term safety.

## Figures and Tables

**Table 1:** Summary of the different studies on stereotactic radiotherapy for ventricular arrhythmia.

Study Year	Patient Number	Sex	Mean Age (Years)	Type of CMP	LVEF (Mean, %)	Dose (Gy)	PTV (Mean, mL)	Treatment Time (Mean, Min)	Delay for Efficacy	Follow-Up (Months)	Complications
Loo et al. [38] 2015	1	M	71	ICMP	24	25	-	90	After 2 months	9	Died from COPD exacerbation at month 9
Cuculich et al. [22] 2017	5	4 M; 1 F	66	2 ICMP; 3 NICMP	23	25	49	14	Progressive effect after ablation, but maximum effect after 6 weeks	12	One fatal stroke 3 weeks after treatment
Jumeau et al. [21] 2018	1	M	75	NICMP	30	25	21	45	Immediate	4	None
Neuwirth et al. [39] 2019	10	9 M; 1 F	66	8 ICMP; 2 NICMP	27	25	22.2	68	Progressive effect	28	Three died of non-arrhythmic causes; progression of mitral valve regurgitation at 17 months
Robinson et al. [40] 2019	19	17 M; 2 F	66	11 ICMP; 5 NICMP; 3 others	25	25	98.9	15	Within the first 6 weeks	13	Pericarditis; heart failure exacerbation at 2 months
Lloyd et al. [41] 2020	10	7 M; 3 F	62	4 ICMP; 4 NICMP; 2 others	-	25	81.4	-	Within the first 2 weeks	6	Mild pneumonitis responsive to corticosteroids in two patients
Gianni et al. [42] 2020	5	5 M	63	4 ICMP; 1 NICMP	34	25	143	82	Four patients had marked reduction in VT burden during first 6 months	12	Two died of heart failure
Chin et al. [43] 2021	8	8 M	75	4 ICMP; 4 NICMP	21	22.2	121.4	18.2	3 months	7.8	No acute complications, three patient deaths in the follow-up period, unrelated to SBRT.
Carbucicchio C [44] 2021	7	8 M	70	3 ICMP; 4 NICMP	27	25	183	31	3 months	8	three patient deaths in the follow-up period, unrelated to SBRT.
Qian et al. [45] 2021	6	6 M	72	6 ICMP	20	25	319	-	6 months	7.7	3 patients died of heart failure; 3 of 6 patients had possible adverse events
Ho et al. [34] 2021	6	6 M	74	2 ICMP; 4 NICMP	29	25	120.3	21.1	-	6	1 patient Pericardial effusion 12 months after therapy
Haskova et al. [46] 2018	1	-	34	NICMP	-	25	-	-	8 months	8	-
Martí Almor et al. [47] 2020	1	M	64	NICMP	-	25	-	4	Immediate	4	None
Scholz et al. [48] 2019	1	M	53	ICMP	-	30	82.4	5/30	2 weeks	2	None
Zeng et al. [49] 2019	1	M	29	NICMP	-	24	71	-	1 month	4	None
Krug et al. [50] 2019	1	M	78	NICMP	15	25	42.2	15	Days	-	Died 57 days after ablation due to sepsis-associated cardiac circulatory failure
Mayinger et al. [51] 2020	1	M	71	NICMP	25	25	115.1	24	48 h	3	None

CMP—cardiomyopathy; COPD—chronic obstructive pulmonary disease; F—female; PTV—planning target volume; ICMP—ischemic cardiomyopathy; LVEF—left ventricular ejection fraction; M—male; NICMP—non-ischemic cardiomyopathy; VT—ventricular tachycardia.
